# 3-(*n*-Propyl­imino­meth­yl)-1,1′-bi-2-naphthol ethanol solvate

**DOI:** 10.1107/S1600536808020436

**Published:** 2008-07-09

**Authors:** Ling-Zhi Zhong, Kun Wang, Xiao-Yan Ma, Rui-Xiang Li

**Affiliations:** a Institute of Homogeneous Catalysis, Department of Chemistry, Sichuan University, Chengdu 610064, People’s Republic of China

## Abstract

In the title compound, C_24_H_21_NO_2_·C_2_H_6_O, the dihedral angle between the two aromatic ring systems is 87.00 (6)°. There is an intra­molecular O—H⋯N hydrogen bond, which forms a six-membered ring. Inter­molecular O—H⋯O hydrogen bonds stabilize the crystal structure.

## Related literature

For background on the application of salen complexes to asymmetric catalysis, see: Pu (1998 ▶). For synthesis, see: Chin *et al.* (2004 ▶).
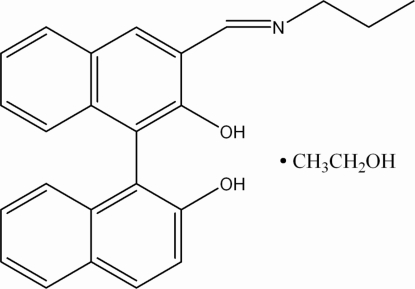


## Experimental

### 

#### Crystal data

C_24_H_21_NO_2_·C_2_H_6_O
*M* *_r_* = 401.49
Triclinic, 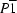

*a* = 10.356 (5) Å
*b* = 10.702 (4) Å
*c* = 11.681 (6) Å
α = 94.74 (3)°
β = 113.53 (4)°
γ = 110.21 (3)°
*V* = 1076.7 (10) Å^3^
*Z* = 2
Mo *K*α radiation
μ = 0.08 mm^−1^
*T* = 292 (2) K
0.42 × 0.40 × 0.38 mm

#### Data collection

Enraf–Nonius CAD-4 diffractometer
Absorption correction: none
3981 measured reflections
3973 independent reflections
1867 reflections with *I* > 2σ(*I*)
*R*_int_ = 0.002
3 standard reflections every 300 reflections intensity decay: 2.1%

#### Refinement

*R*[*F* ^2^ > 2σ(*F* ^2^)] = 0.045
*wR*(*F* ^2^) = 0.120
*S* = 0.94
3973 reflections
276 parameters
H-atom parameters constrained
Δρ_max_ = 0.20 e Å^−3^
Δρ_min_ = −0.16 e Å^−3^

### 

Data collection: *DIFRAC* (Gabe & White, 1993 ▶); cell refinement: *DIFRAC*; data reduction: *NRCVAX* (Gabe *et al.*, 1989 ▶); program(s) used to solve structure: *SHELXS97* (Sheldrick, 2008 ▶); program(s) used to refine structure: *SHELXL97* (Sheldrick, 2008 ▶); molecular graphics: *SHELXTL* (Sheldrick, 2008 ▶); software used to prepare material for publication: *SHELXTL*.

## Supplementary Material

Crystal structure: contains datablocks I, global. DOI: 10.1107/S1600536808020436/bt2737sup1.cif

Structure factors: contains datablocks I. DOI: 10.1107/S1600536808020436/bt2737Isup2.hkl

Additional supplementary materials:  crystallographic information; 3D view; checkCIF report

## Figures and Tables

**Table 1:** Hydrogen-bond geometry (Å, °)

*D*—H⋯*A*	*D*—H	H⋯*A*	*D*⋯*A*	*D*—H⋯*A*
O1—H1⋯O3^i^	0.82	1.92	2.738 (2)	175
O2—H2⋯N1	0.82	1.85	2.590 (3)	149
O3—H3⋯O2	0.82	2.19	2.939 (3)	151

Symmetry code: (i) 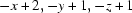.

**Fig. 1.**
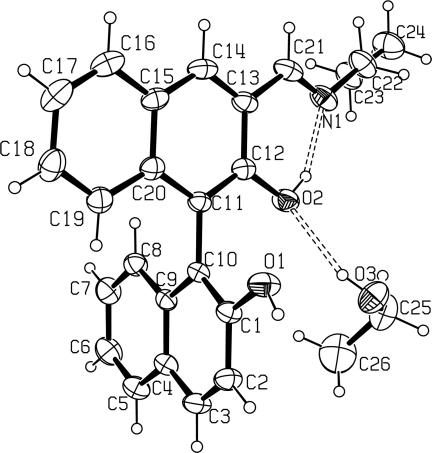
A perspective view of the title compound.

**Fig. 2.**
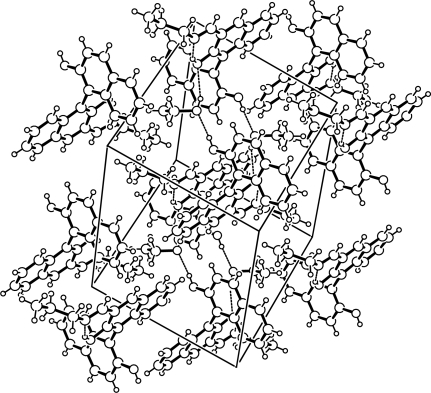
Hydrogen bonding in the crystal structure of the title compound.

## supplementary crystallographic information

### Comment

Binol and its derivatives have been largely used in asymmetric catalysis and chiral recognition (Pu, 1998). In this paper we present X-ray crystallographic analysis of the title compound .

As shown in Fig. 1, an intramolecular O—H···N hydrogen bond between the hydroxy and the imino moieties forms a ring.

In the crystal, the molecules are connected by O—H···O hydrogen bonds (Fig. 2).

### Experimental

The salen ligand, 3-(n-propyliminomethyl)-1,1'-binaphthol was prepared by condensation of 3-carboxaldehyde-1,1'-binaphthol with *n*-propyamine, which was prepared by reported methods (Chin *et al.*, 2004). Crystals suitable for X-ray analysis were obtained by slow evaporation of a ethanol /methylene chloride (1:5) solution of the compound.

### Refinement

All   H atoms were placed in calculated positions and refined using a riding-model with C-H ranging from 0.93 to 0.97Å and O-H = 0.82Å and U(H)= 1.2U_eq_(C,O) or U(H)= 1.5U_eq_(C_methyl_). The methyl and hydroxyl groups were allowed to rotate but not to tip.

### Crystal data

**Table:**

C_24_H_21_NO_2_·C_2_H_6_O	*Z* = 2
*M**_r_* = 401.49	*F*_000_ = 428
Triclinic, *P*1	*D*_x_ = 1.239 Mg m^−^^3^
Hall symbol: -P 1	Mo *K*α radiation λ = 0.71073 Å
*a* = 10.356 (5) Å	Cell parameters from 37 reflections
*b* = 10.702 (4) Å	θ = 4.6–9.7º
*c* = 11.681 (6) Å	µ = 0.08 mm^−^^1^
α = 94.74 (3)º	*T* = 292 (2) K
β = 113.53 (4)º	Block, red
γ = 110.21 (3)º	0.42 × 0.40 × 0.38 mm
*V* = 1076.7 (10) Å^3^	

### Data collection

**Table:**

Enraf–Nonius CAD-4 diffractometer	*R*_int_ = 0.002
Radiation source: fine-focus sealed tube	θ_max_ = 25.4º
Monochromator: graphite	θ_min_ = 2.0º
*T* = 291(2) K	*h* = −12→11
ω/2–θ scans	*k* = −4→12
Absorption correction: none	*l* = −14→14
3981 measured reflections	3 standard reflections
3973 independent reflections	every 300 reflections
1867 reflections with *I* > 2σ(*I*)	intensity decay: 2.1%

### Refinement

**Table:**

Refinement on *F*^2^	Hydrogen site location: inferred from neighbouring sites
Least-squares matrix: full	H-atom parameters constrained
*R*[*F*^2^ > 2σ(*F*^2^)] = 0.045	*w* = 1/[σ^2^(*F*_o_^2^) + (0.0567*P*)^2^] where *P* = (*F*_o_^2^ + 2*F*_c_^2^)/3
*wR*(*F*^2^) = 0.120	(Δ/σ)_max_ < 0.001
*S* = 0.94	Δρ_max_ = 0.20 e Å^−^^3^
3973 reflections	Δρ_min_ = −0.16 e Å^−^^3^
276 parameters	Extinction correction: SHELXL97 (Sheldrick, 2008), Fc^*^=kFc[1+0.001xFc^2^λ^3^/sin(2θ)]^-1/4^
Primary atom site location: structure-invariant direct methods	Extinction coefficient: 0.039 (3)
Secondary atom site location: difference Fourier map	

### Special details

**Table:**

Geometry. All e.s.d.'s (except the e.s.d. in the dihedral angle between two l.s. planes) are estimated using the full covariance matrix. The cell e.s.d.'s are taken into account individually in the estimation of e.s.d.'s in distances, angles and torsion angles; correlations between e.s.d.'s in cell parameters are only used when they are defined by crystal symmetry. An approximate (isotropic) treatment of cell e.s.d.'s is used for estimating e.s.d.'s involving l.s. planes.
Refinement. Refinement of *F*^2^ against ALL reflections. The weighted *R*-factor *wR* and goodness of fit *S* are based on *F*^2^, conventional *R*-factors *R* are based on *F*, with *F* set to zero for negative *F*^2^. The threshold expression of *F*^2^ > 2σ(*F*^2^) is used only for calculating *R*-factors(gt) *etc*. and is not relevant to the choice of reflections for refinement. *R*-factors based on *F*^2^ are statistically about twice as large as those based on *F*, and *R*- factors based on ALL data will be even larger.

### Fractional atomic coordinates and isotropic or equivalent isotropic displacement parameters (Å^2^)

**Table:**

	*x*	*y*	*z*	*U*_iso_*/*U*_eq_	
O1	1.05783 (17)	0.49721 (14)	0.36028 (17)	0.0594 (5)	
H1	1.0117	0.5411	0.3732	0.071*	
O2	1.23674 (17)	0.25319 (17)	0.46653 (16)	0.0542 (5)	
H2	1.3092	0.2426	0.5213	0.065*	
O3	1.0938 (2)	0.34869 (17)	0.60697 (19)	0.0706 (6)	
H3	1.1196	0.3381	0.5503	0.085*	
N1	1.5128 (2)	0.2638 (2)	0.5883 (2)	0.0577 (6)	
C1	0.9549 (2)	0.3634 (2)	0.2954 (2)	0.0422 (6)	
C2	0.8018 (3)	0.3178 (2)	0.2763 (2)	0.0499 (6)	
H2A	0.7694	0.3791	0.3055	0.060*	
C3	0.7007 (3)	0.1847 (2)	0.2154 (2)	0.0530 (7)	
H3A	0.5997	0.1553	0.2039	0.064*	
C4	0.7464 (2)	0.0903 (2)	0.1693 (2)	0.0438 (6)	
C5	0.6427 (3)	−0.0497 (2)	0.1053 (2)	0.0574 (7)	
H5	0.5418	−0.0811	0.0946	0.069*	
C6	0.6889 (3)	−0.1383 (2)	0.0595 (2)	0.0631 (8)	
H6	0.6194	−0.2294	0.0169	0.076*	
C7	0.8399 (3)	−0.0930 (2)	0.0762 (2)	0.0571 (7)	
H7	0.8705	−0.1542	0.0444	0.069*	
C8	0.9434 (3)	0.0399 (2)	0.1384 (2)	0.0470 (6)	
H8	1.0439	0.0681	0.1488	0.056*	
C9	0.9004 (2)	0.1357 (2)	0.1875 (2)	0.0379 (5)	
C10	1.0073 (2)	0.2762 (2)	0.25333 (19)	0.0365 (5)	
C11	1.1704 (2)	0.3247 (2)	0.2740 (2)	0.0375 (5)	
C12	1.2792 (2)	0.3084 (2)	0.3796 (2)	0.0408 (6)	
C13	1.4355 (2)	0.3485 (2)	0.4005 (2)	0.0445 (6)	
C14	1.4773 (3)	0.4108 (2)	0.3151 (2)	0.0511 (7)	
H14	1.5796	0.4407	0.3302	0.061*	
C15	1.3714 (3)	0.4312 (2)	0.2059 (2)	0.0461 (6)	
C16	1.4142 (3)	0.4958 (2)	0.1180 (3)	0.0625 (7)	
H16	1.5173	0.5310	0.1345	0.075*	
C17	1.3077 (4)	0.5073 (3)	0.0103 (3)	0.0691 (8)	
H17	1.3382	0.5510	−0.0460	0.083*	
C18	1.1518 (3)	0.4538 (3)	−0.0169 (3)	0.0625 (7)	
H18	1.0785	0.4583	−0.0933	0.075*	
C19	1.1064 (3)	0.3949 (2)	0.0678 (2)	0.0490 (6)	
H19	1.0027	0.3619	0.0493	0.059*	
C20	1.2138 (2)	0.3833 (2)	0.1827 (2)	0.0418 (6)	
C21	1.5484 (3)	0.3257 (2)	0.5097 (3)	0.0539 (7)	
H21	1.6501	0.3574	0.5226	0.065*	
C22	1.6320 (3)	0.2426 (2)	0.6952 (2)	0.0657 (8)	
H22A	1.6428	0.2869	0.7764	0.079*	
H22B	1.7303	0.2855	0.6937	0.079*	
C23	1.5938 (3)	0.0939 (3)	0.6875 (2)	0.0654 (8)	
H23A	1.5885	0.0510	0.6083	0.078*	
H23B	1.4925	0.0502	0.6837	0.078*	
C24	1.7112 (3)	0.0693 (3)	0.8015 (3)	0.0837 (9)	
H24A	1.8123	0.1147	0.8074	0.126*	
H24B	1.6842	−0.0278	0.7895	0.126*	
H24C	1.7115	0.1055	0.8796	0.126*	
C25	1.0527 (4)	0.2247 (3)	0.6459 (3)	0.0819 (9)	
H25A	1.0438	0.2428	0.7246	0.098*	
H25B	1.1341	0.1927	0.6647	0.098*	
C26	0.9048 (4)	0.1156 (3)	0.5454 (3)	0.1148 (13)	
H26A	0.8227	0.1446	0.5305	0.172*	
H26B	0.8834	0.0325	0.5739	0.172*	
H26C	0.9123	0.0988	0.4668	0.172*	

### Atomic displacement parameters (Å^2^)

**Table:**

	*U*^11^	*U*^22^	*U*^33^	*U*^12^	*U*^13^	*U*^23^
O1	0.0426 (10)	0.0422 (9)	0.0837 (13)	0.0137 (8)	0.0270 (9)	−0.0041 (9)
O2	0.0418 (10)	0.0671 (10)	0.0566 (12)	0.0264 (9)	0.0201 (9)	0.0260 (9)
O3	0.0925 (15)	0.0585 (11)	0.0825 (15)	0.0393 (11)	0.0532 (12)	0.0186 (10)
N1	0.0453 (13)	0.0549 (13)	0.0607 (15)	0.0258 (11)	0.0092 (11)	0.0122 (11)
C1	0.0345 (13)	0.0363 (12)	0.0501 (15)	0.0130 (11)	0.0161 (11)	0.0073 (11)
C2	0.0412 (14)	0.0496 (15)	0.0622 (17)	0.0217 (12)	0.0250 (12)	0.0099 (12)
C3	0.0330 (13)	0.0586 (16)	0.0629 (17)	0.0177 (12)	0.0194 (12)	0.0116 (13)
C4	0.0338 (13)	0.0431 (13)	0.0457 (15)	0.0125 (11)	0.0132 (11)	0.0096 (11)
C5	0.0389 (14)	0.0526 (15)	0.0619 (17)	0.0091 (13)	0.0153 (13)	0.0083 (13)
C6	0.0558 (18)	0.0429 (15)	0.0629 (19)	0.0072 (14)	0.0147 (14)	0.0022 (13)
C7	0.0617 (18)	0.0452 (15)	0.0572 (17)	0.0232 (13)	0.0211 (14)	0.0044 (12)
C8	0.0443 (14)	0.0472 (14)	0.0486 (15)	0.0222 (12)	0.0182 (12)	0.0090 (12)
C9	0.0346 (13)	0.0406 (12)	0.0358 (13)	0.0162 (10)	0.0128 (10)	0.0107 (10)
C10	0.0290 (12)	0.0390 (12)	0.0369 (13)	0.0137 (10)	0.0112 (10)	0.0090 (10)
C11	0.0322 (12)	0.0351 (12)	0.0433 (14)	0.0149 (10)	0.0157 (11)	0.0056 (10)
C12	0.0390 (13)	0.0346 (12)	0.0485 (15)	0.0140 (10)	0.0213 (12)	0.0085 (11)
C13	0.0343 (13)	0.0403 (13)	0.0551 (16)	0.0180 (11)	0.0162 (12)	0.0054 (11)
C14	0.0366 (14)	0.0458 (14)	0.0752 (19)	0.0181 (12)	0.0298 (14)	0.0092 (13)
C15	0.0461 (15)	0.0442 (13)	0.0601 (17)	0.0235 (12)	0.0313 (13)	0.0133 (12)
C16	0.0649 (18)	0.0616 (17)	0.086 (2)	0.0319 (15)	0.0522 (17)	0.0241 (15)
C17	0.092 (2)	0.0770 (19)	0.080 (2)	0.0509 (18)	0.0620 (19)	0.0361 (16)
C18	0.085 (2)	0.0717 (18)	0.0572 (18)	0.0507 (17)	0.0407 (16)	0.0227 (15)
C19	0.0524 (15)	0.0507 (14)	0.0504 (16)	0.0271 (12)	0.0246 (13)	0.0130 (12)
C20	0.0439 (14)	0.0368 (12)	0.0488 (15)	0.0199 (11)	0.0232 (12)	0.0060 (11)
C21	0.0342 (14)	0.0452 (15)	0.0705 (19)	0.0178 (12)	0.0133 (13)	0.0060 (13)
C22	0.0535 (16)	0.0645 (17)	0.0633 (18)	0.0300 (14)	0.0085 (14)	0.0109 (14)
C23	0.0583 (17)	0.0684 (17)	0.0583 (17)	0.0280 (14)	0.0137 (14)	0.0221 (13)
C24	0.0665 (19)	0.096 (2)	0.088 (2)	0.0434 (17)	0.0230 (17)	0.0439 (18)
C25	0.123 (3)	0.077 (2)	0.074 (2)	0.056 (2)	0.056 (2)	0.0300 (17)
C26	0.122 (3)	0.078 (2)	0.123 (3)	0.015 (2)	0.059 (3)	0.025 (2)

### Geometric parameters (Å, °)

**Table:**

O1—C1	1.372 (2)	C13—C21	1.453 (3)
O1—H1	0.8200	C14—C15	1.404 (3)
O2—C12	1.360 (3)	C14—H14	0.9300
O2—H2	0.8200	C15—C16	1.414 (3)
O3—C25	1.419 (3)	C15—C20	1.430 (3)
O3—H3	0.8200	C16—C17	1.350 (4)
N1—C21	1.272 (3)	C16—H16	0.9300
N1—C22	1.464 (3)	C17—C18	1.399 (4)
C1—C10	1.376 (3)	C17—H17	0.9300
C1—C2	1.403 (3)	C18—C19	1.367 (3)
C2—C3	1.356 (3)	C18—H18	0.9300
C2—H2A	0.9300	C19—C20	1.409 (3)
C3—C4	1.406 (3)	C19—H19	0.9300
C3—H3A	0.9300	C21—H21	0.9300
C4—C9	1.415 (3)	C22—C23	1.486 (3)
C4—C5	1.421 (3)	C22—H22A	0.9700
C5—C6	1.359 (3)	C22—H22B	0.9700
C5—H5	0.9300	C23—C24	1.519 (3)
C6—C7	1.391 (3)	C23—H23A	0.9700
C6—H6	0.9300	C23—H23B	0.9700
C7—C8	1.362 (3)	C24—H24A	0.9600
C7—H7	0.9300	C24—H24B	0.9600
C8—C9	1.413 (3)	C24—H24C	0.9600
C8—H8	0.9300	C25—C26	1.480 (4)
C9—C10	1.435 (3)	C25—H25A	0.9700
C10—C11	1.493 (3)	C25—H25B	0.9700
C11—C12	1.372 (3)	C26—H26A	0.9600
C11—C20	1.424 (3)	C26—H26B	0.9600
C12—C13	1.433 (3)	C26—H26C	0.9600
C13—C14	1.371 (3)		
			
C1—O1—H1	109.5	C17—C16—C15	121.1 (3)
C12—O2—H2	109.5	C17—C16—H16	119.5
C25—O3—H3	109.5	C15—C16—H16	119.5
C21—N1—C22	119.3 (2)	C16—C17—C18	120.3 (3)
O1—C1—C10	118.24 (19)	C16—C17—H17	119.9
O1—C1—C2	119.98 (19)	C18—C17—H17	119.9
C10—C1—C2	121.76 (19)	C19—C18—C17	120.5 (3)
C3—C2—C1	120.2 (2)	C19—C18—H18	119.8
C3—C2—H2A	119.9	C17—C18—H18	119.8
C1—C2—H2A	119.9	C18—C19—C20	121.2 (2)
C2—C3—C4	121.0 (2)	C18—C19—H19	119.4
C2—C3—H3A	119.5	C20—C19—H19	119.4
C4—C3—H3A	119.5	C19—C20—C11	122.5 (2)
C3—C4—C9	119.16 (19)	C19—C20—C15	117.8 (2)
C3—C4—C5	122.0 (2)	C11—C20—C15	119.7 (2)
C9—C4—C5	118.9 (2)	N1—C21—C13	122.2 (2)
C6—C5—C4	120.8 (2)	N1—C21—H21	118.9
C6—C5—H5	119.6	C13—C21—H21	118.9
C4—C5—H5	119.6	N1—C22—C23	111.9 (2)
C5—C6—C7	120.2 (2)	N1—C22—H22A	109.2
C5—C6—H6	119.9	C23—C22—H22A	109.2
C7—C6—H6	119.9	N1—C22—H22B	109.2
C8—C7—C6	120.8 (2)	C23—C22—H22B	109.2
C8—C7—H7	119.6	H22A—C22—H22B	107.9
C6—C7—H7	119.6	C22—C23—C24	112.9 (2)
C7—C8—C9	121.0 (2)	C22—C23—H23A	109.0
C7—C8—H8	119.5	C24—C23—H23A	109.0
C9—C8—H8	119.5	C22—C23—H23B	109.0
C8—C9—C4	118.3 (2)	C24—C23—H23B	109.0
C8—C9—C10	122.0 (2)	H23A—C23—H23B	107.8
C4—C9—C10	119.63 (19)	C23—C24—H24A	109.5
C1—C10—C9	118.25 (19)	C23—C24—H24B	109.5
C1—C10—C11	121.72 (18)	H24A—C24—H24B	109.5
C9—C10—C11	120.02 (18)	C23—C24—H24C	109.5
C12—C11—C20	119.3 (2)	H24A—C24—H24C	109.5
C12—C11—C10	119.8 (2)	H24B—C24—H24C	109.5
C20—C11—C10	120.84 (19)	O3—C25—C26	112.0 (3)
O2—C12—C11	118.8 (2)	O3—C25—H25A	109.2
O2—C12—C13	119.5 (2)	C26—C25—H25A	109.2
C11—C12—C13	121.7 (2)	O3—C25—H25B	109.2
C14—C13—C12	118.3 (2)	C26—C25—H25B	109.2
C14—C13—C21	120.4 (2)	H25A—C25—H25B	107.9
C12—C13—C21	121.4 (2)	C25—C26—H26A	109.5
C13—C14—C15	122.5 (2)	C25—C26—H26B	109.5
C13—C14—H14	118.7	H26A—C26—H26B	109.5
C15—C14—H14	118.7	C25—C26—H26C	109.5
C14—C15—C16	122.7 (2)	H26A—C26—H26C	109.5
C14—C15—C20	118.3 (2)	H26B—C26—H26C	109.5
C16—C15—C20	119.0 (2)		

### Hydrogen-bond geometry (Å, °)

**Table:**

*D*—H···*A*	*D*—H	H···*A*	*D*···*A*	*D*—H···*A*
O1—H1···O3^i^	0.82	1.92	2.738 (2)	175
O2—H2···N1	0.82	1.85	2.590 (3)	149
O3—H3···O2	0.82	2.19	2.939 (3)	151

Symmetry codes: (i) −*x*+2, −*y*+1, −*z*+1.
